# Identification of functional pathways and molecular signatures in neuroendocrine neoplasms by multi-omics analysis

**DOI:** 10.1186/s12967-022-03511-7

**Published:** 2022-07-06

**Authors:** Viola Melone, Annamaria Salvati, Domenico Palumbo, Giorgio Giurato, Giovanni Nassa, Francesca Rizzo, Luigi Palo, Alessandro Giordano, Mariarosaria Incoronato, Mario Vitale, Caterina Mian, Immacolata Di Biase, Stefano Cristiano, Viviana Narciso, Monica Cantile, Annabella Di Mauro, Fabiana Tatangelo, Salvatore Tafuto, Roberta Modica, Claudia Pivonello, Marco Salvatore, Annamaria Colao, Alessandro Weisz, Roberta Tarallo

**Affiliations:** 1 Laboratory of Molecular Medicine and Genomics, Department of Medicine, Surgery and Dentistry “Scuola Medica Salernitana”, University of Salerno, via S. Allende, 84081 Baronissi, SA Italy; 2 Medical Genomics Program and Division of Oncology, AOU ‘S. Giovanni di Dio e Ruggi d’Aragona’ University of Salerno, Rete Oncologica Campana, 84131 Salerno, Italy; 3 Genome Research Center for Health, 84081 Baronissi, SA Italy; 4 IRCCS Synlab SDN s.p.a, Via Gianturco 113, 80143 Naples, Italy; 5 Department of Medicine, Surgery and Dentistry “Scuola Medica Salernitana”, University of Salerno, 84081 Baronissi, SA Italy; 6 Endocrinology Unit, Department of Medicine (DIMED), University of Padua, Padua, Italy; 7 MeriGen Diagnostic & c sas, traversa M. Pietravalle 11, 80131 Naples, Italy; 8 Pathology Unit, Istituto Nazionale Tumori-IRCCS-Fondazione G. Pascale, Via Mariano Semmola, 80131 Naples, Italy; 9 Sarcomas and Rare Tumors Unit, Istituto Nazionale Tumori-IRCCS-Fondazione G. Pascale, Via Mariano Semmola, 80131 Naples, Italy; 10 Department of Clinical Medicine and Surgery, Endocrinology Unit, Federico II University, Naples, Italy; 11 UNESCO Chair for Health Education and Sustainable Development, Federico II University, Naples, Italy

**Keywords:** Neuroendocrine neoplasms, Molecular signatures, ATM signaling, Circulating biomarkers

## Abstract

**Background:**

Neuroendocrine neoplasms (NENs) represent a heterogeneous class of rare tumors with increasing incidence. They are characterized by the ability to secrete peptide hormones and biogenic amines but other reliable biomarkers are lacking, making diagnosis and identification of the primary site very challenging. While in some NENs, such as the pancreatic ones, next generation sequencing technologies allowed the identification of new molecular hallmarks, our knowledge of the molecular profile of NENs from other anatomical sites is still poor.

**Methods:**

Starting from the concept that NENs from different organs may be clinically and genetically correlated, we applied a multi-omics approach by combining multigene panel testing, CGH-array, transcriptome and miRNome profiling and computational analyses, with the aim to highlight common molecular and functional signatures of gastroenteropancreatic (GEP)-NENs and medullary thyroid carcinomas (MTCs) that could aid diagnosis, prognosis and therapy.

**Results:**

By comparing genomic and transcriptional profiles, ATM-dependent signaling emerged among the most significant pathways at multiple levels, involving gene variations and miRNA-mediated regulation, thus representing a novel putative druggable pathway in these cancer types. Moreover, a set of circulating miRNAs was also selected as possible diagnostic/prognostic biomarkers useful for clinical management of NENs.

**Conclusions:**

These findings depict a complex molecular and functional landscape of NENs, shedding light on novel therapeutic targets and disease biomarkers to be exploited.

**Supplementary Information:**

The online version contains supplementary material available at 10.1186/s12967-022-03511-7.

## Introduction

Neuroendocrine neoplasms (NENs) are a class of rare and heterogeneous tumors whose molecular pathogenesis is an open issue. NENs are characterized by a body-wide distribution because they develop from neuroendocrine system cells, which are spread throughout the whole body [1–3]; they mainly arise in gastrointestinal and pulmonary tract, but can also arise from thyroid, pituitary gland, lung, breast or larynx or other organs and tissues [4]. These neoplasms can occur both in sporadic form and in hereditary syndromes such as multiple endocrine neoplasia type 1 and 2 (MEN1 and MEN2), von Hippel–Lindau disease (VHL), neurofibromatosis type 1 (NF1) and the tuberous sclerosis complex (TSC) [5, 6].

NENs originating from pancreas and the gastro-intestinal tract, the gastroenteropancreatic NENs (GEP-NENs) are among the most common forms. The World Health Organization (WHO) and the International Agency for Research on Cancer (IARC) classified GEP-NENs based on tumor primary sites and on the morphological differentiation features by which these neoplasms can be divided into the well-differentiated tumors (NETs) and poorly-differentiated carcinomas (NECs) [4]. According to proliferation index and mitotic count, GEP-NENs have been categorized into low (G1, Ki67 < 3%), intermediate (G2, Ki67 3–20%) and high (G3, Ki67 > 20%) grades [7]. Particularly, NET comprised well differentiated tumors with G1, G2 and G3 grade, while NEC comprised poorly differentiated carcinomas with G3 grade [7].

The thyroid NENs are tumors of parafollicular C-cells that are conventionally known as medullary thyroid carcinomas (MTCs) [8]; they represent 3–5% of all thyroid carcinomas and can develop, in ~ 30% of the cases, in the context of MEN2 syndromes [9]. In MTCs the Ki-67 index, conventionally used for GEP-NENs classification, is difficult to assess, being often lower than 1%, so a classification based on this parameter is not currently used [4].

Specific genomic profiles and genetic signatures have been previously observed among GEP-NENs with different primary sites and degrees of differentiation and in MTCs, with pancreatic NENs being the best described in the literature. In pancreatic NETs, somatic mutations in MEN1, DAXX, ATRX, PTEN, TSC2 and members of the mTOR signaling pathway were observed [10–12]. Moreover, sporadic pancreatic NETs also present germline mutations in the DNA repair genes MUTYH, CHEK2 and BRCA2 [11]. On the other hand, gastrointestinal NETs (GI-NETs) frequently show mutations in CDNK1B gene [13, 14]. In contrast, both pancreatic and intestinal NECs commonly show mutations in TP53 and RB1 and may share mutations in KRAS and SMAD4 [13, 15, 16]. In MTCs mutations in RET gene were described, affecting tumor microenvironment and angiogenesis, and this has been linked to poor prognosis compared to MTCs that are RAS mutated [4, 17]. Overall, from the genomic point of view, the loss of chromosome 18 has been reported in small bowel NETs, even if the biological significance of this alteration is still unknown [18]. However, in pancreatic NETs, the loss of genetic material has been described more often than chromosomal gains [11].

Generally, NENs are characterized by a relatively indolent rate of growth and by the ability to secrete peptide hormones and biogenic amines that are used as biomarkers [18]. However, over the latest 40 years the incidence and the prevalence of these tumors have increased more than sixfold in the United States [19] and, due to non-specific symptomatology and lack of early markers, many NENs show metastatic profile at diagnosis, making it sometimes impossible to identify the primary site of tumor lesion [3, 20, 21].

A further problem, in addition to a more accurate classification, is the lack of specific markers for NEN diagnosis; Chromogranin A (CgA), synaptophysin (Syn), 5-Hydroxyindoleacetic Acid (5-HIAA), neuron-specific enolase (NSE) and cluster of differentiation 56 (CD56) (neural cell adhesion molecule) are currently used for GEP-NENs diagnosis [18, 22] and Calcitonin for MTCs [23]. In GEP-NENs, both Syn and CgA are highly expressed in well-differentiated neoplasms, whereas poorly differentiated carcinomas often maintain synaptophysin positivity while losing CgA expression and acquiring NSE expression [18]. CgA is characterized by low sensitivity and specificity and the tests can give lots of false-positive elevations [24, 25]. Equally, the prognostic role of 5-HIAA remains controversial [26]. For MTC diagnosis, calcitonin is a sensitive tumor marker because it correlates with C-cell mass and burden of the neoplasms [23], but this has also some limitations, such as inter-assay variability, concentration-dependent half-life and rapid degradation [23].

To meet this clinical need, in our study we aimed to identify novel prognostic factors and biomarkers for the improvement of the histologic and pathologic evaluation of NENs which is a key component of clinical management [27, 28]. Particularly, our attention was focused on genome, transcriptome and miRNome profiles of tumor biopsies through a multi-omics approach. The case cohort studied included 66 specimens from GEP-NENs at different grades and MTCs. In few cases only metastatic tissues were available, mainly among neoplasms with gastroenteropancreatic primary location, and these were analyzed as a separate group. Moreover, in order to link our results to clinical management, serum samples of NENs patients were analyzed to determine presence and concentration in the serum of NEN patients of the miRNAs highly expressed within the corresponding tumor tissue. This study design allowed the identification of a subset of molecules able to discriminate healthy from sick subjects, as well as to find some miRNAs significantly correlating with clinical-pathologic features of the neoplasms. These might have a strong impact for diagnostic and prognostic purposes respectively, as therapeutic sequence in patients with NENs is still debated [29, 30].

Moreover, altogether the obtained results revealed the ataxia telangiectasia mutated (ATM) signaling among the most significantly impacted at different levels, considering gene variants as mutations and amplifications and miRNA expression deregulation. Indeed, this might represent a putative targetable pathway in the treatment of NENs.

## Subjects and methods

### Patients characteristics and pathological assessment

Tumor biopsies from 46 NEN patients (Thyroid n = 17, Pancreas n = 14, Intestine n = 12 and Lung n = 3) were collected by the biobank of the “Istituto Nazionale Tumori-IRCCS-Fondazione G. Pascale” (Naples, Italy) and by the Department of Clinical Medicine and Surgery, Endocrinology Unit of Federico II University (Naples, Italy). Out of 46 tumor tissues, 19 were FFPE sections (Formalin fixed paraffin embedded) while 27 were frozen sections. In addition, a validation set of 20 previously isolated RNAs from MTCs were obtained from Endocrinology Unit, Department of Medicine (DIMED), University of Padua. Patient characteristics and samples pathology were summarized in Table 1.

**Table 1:** Patients’ clinical data

Classification	Number/percentage (n = 66)
Gender	
Male	33 (50%)
Female	33 (50%)
Tumor location	
Thyroid	37 (56.1%)
Pancreas	14 (21.2%)
Intestine	12 (18.2%)
Lung	3 (4.5%)
Metastases	19 (28.8%)
Grading classification (WHO 2019) for p-NET, I-NET and lung-NET	
NET G1 (Ki-67% ≤ 2)	11 (38%)
NET G2 (Ki-67% 3–20)	11 (38%)
NEC G3 (Ki-67% > 20)	7 (24%)
Genetic syndrome	
Men1	1 (2%)
Men2	3 (7%)
ND	1 (2%)
Status	
Live	53 (80.3%)
Dead	7 (10.6%)
nd/progression	6 (9.1%)

Cases have been reviewed by an expert pathologist (FT) and graded and staged according to WHO 2017 and 2019 classification criteria (NET-G1, NET-G2, NET-G3, NEC-G3) on tissue sections. The 4 main categories are distinguished on the basis of the proliferative activity, measured through the mitotic count and the Ki67 expression. In our cohort, high-grade (G3) specimen were all classified as poorly differentiated according to Hematoxylin/Eosin staining, thus they all fall within NECs. Medical records have been reviewed for clinical information, including histologic parameters, assessed on standard H&E-stained slides combined with immunohistochemical staining with neuroendocrine markers, and tumor location. Immunohistochemical staining for Ki67 (clone MM1, Leica), Chromogranin A (clone 5H7, Leica, Wetzlar, Germany, ready to use), Synaptophysin (clone 27G12, Leica, ready to use) and Calcitonin (clone CL1948, Leica, ready to use) has been performed using the En Vision method (DAKO, Denmark) following the manufacturer’s instruction.

For miRNA validation in liquid biopsies, serum from 42 NEN patients (6 MTCs and 36 GEP-NENs) and 34 healthy subjects were obtained.

### Nucleic acids extraction

DNA and RNA isolations were performed from both FFPE and frozen sections using FFPE DNA Purification Kit (Cat. 47400, Norgen Biotek Corp, Thorold, Canada) and RNA/DNA Purification Kit (Cat. 48700, Norgen Biotek Corp.) respectively, according to the manufacturers protocol. Nucleic acids were quantified with Qubit 2.0 fluorometer using Qubit RNA HS assay kit and Qubit DNA HS assay kit (Thermo Fisher Scientific, Waltham, Massachusetts, USA). The assessment of nucleic acids integrity (DIN and RIN) was performed with Agilent 4150 TapeStation System (Agilent Technologies, Santa Clara, CA, USA).

### Mutational profiling

Libraries for mutational profiling were prepared, starting from 40 ng of DNA as input material, with TruSight™ Oncology500 kit (Cat. 20028213, Illumina, San Diego, California, USA) according to manufacturer’s protocol. These consist in a targeted-capture of 523 cancer-relevant genes. The libraries were sequenced on NextSeq 500 (Illumina) in 2 × 150 bp or 2 × 75 bp in paired end mode. Sequencing data were analyzed using the TruSight Oncology 500 Local App Version 2.2 (Illumina) to identify variants, gene amplifications, TMB and MSI. The genomic coordinates of all the identified variants were subsequently converted to hg38 using LiftOver [31].

Variants were annotated with Annovar [32]. Those with coverage depth lower than 100 and variants occurring with a frequency higher than 5% in 1000G or GnomAD were discarded. The sequence variants annotated as “benign” or “likely benign” in ClinVar database were also filtered out. Furthermore, variants classified as synonymous or in intergenic positions were discarded. Oncoplots were generated using Maftools [33] on R (v4.0.2). Functional analysis was performed using Ingenuity Pathway Analysis (IPA, Qiagen, Hilden, Germany) and only the pathways with more than 1.3 in –log of the adjusted p-value were considered.

### Array-CGH

Array-CGH analysis was performed using Agilent Oligonucleotide Array-Based CGH for Genomic DNA Analyis-Enzimatic Labeling (Agilent Technologies, Santa Clara, CA, USA), starting from 500 ng of DNA, following the manufacturer’s instructions. The microarray includes 60.000 oligonucleotide probes. Genomic DNA samples and reference samples were labeled with Cy5 and Cy3, respectively, using the SureTag DNA Labeling Kit and following Agilent Enzymatic Labeling protocol. Labeled genomic DNA was purified using the reaction Purification Column provided with the kit. After the hybridization protocol, slides were scanned using Agilent SureScan Dx Microarray Scanner G5761A. Image files were analyzed using Agilent Cytogenomics 5.0.0.38 Software, and genomic coordinates were evaluated according to GRCh38/hg38. The measure of success of profiling for each sample was based on array data sample quality indices (derivative log ratio scores). Circos plot was generated using circos (v0.69.9) [34].

### Trancriptome profiling

Libraries preparation for transcriptome analysis was performed employing the TruSeq RNA Exome kit (Cat. 20020189, Illumina) for FFPE samples and TruSeq Stranded Total RNA kit (Cat. 20020598, Illumina) for frozen samples, starting from 200 and 400 ng of RNA as input materials, respectively, according to manufacturers’ guidelines. 46 libraries were sequenced on NextSeq 500 (Illumina) using 2 × 75pb paired end and 14 libraries on HiSeq 1500 (Illumina) using 1 × 50 single read.

Raw reads were pre-processed using FastQC software [35] to evaluate raw sequences quality and adapter sequences were removed using cutadapt (v3.3) [36]. Alignment was performed on human genome version hg38 (release 34) with STAR (v2.7.8a) [37] and expressed transcripts were identified using featureCounts on Rsubread (v2.0.1) [38]. Differential expression analysis was performed using DESeq2 package (v1.28.1) in R [39], with default parameters. Transcripts were considered differentially expressed if they showed |FC| ≥ 1.5 and adjusted p-value ≤ 0.05. Fusion transcripts detection was performed with STAR-Fusion tool [40], setting default parameters and only fusion transcripts with more than 10 junction reads were considered.

### Small-RNA profiling

Small-RNA libraries were prepared with NEXTFLEX Small RNA-Seq Kit v3 (Cat NOVA-5132-06, Perkin Elmer, Massachusetts, USA) starting from 50 ng of RNA as input, according to manufacturer’s guidelines. The libraries were sequenced on NextSeq 500 (Illumina) using 1 × 75 bp single read. miRNA-Seq data analysis was performed using the automated pipeline iSmaRT [41]. Target prediction was performed using miRWalk [42]. Only targets validated and present on TargetScan or miRDB with more than 10 reads in at least 60% of the samples were considered. Gene ontology plot was generated using the library GOplot on R [43]. Network was generated using Cytoskape v 3.9.0 [44].

### Serum sampling, RNA extraction, reverse transcription and real-time PCR

Serum was obtained from whole blood samples by centrifugation at 1900×*g* for 10 min at 4 °C. The supernatant was further centrifuged at 16,000×*g* for 10 min at 4 °C and stored in aliquots of 0.5 ml at − 80 °C until analysis. The extraction of total RNA from 200 µl of serum was performed within 1 year of storage at − 80 °C using miRNeasy Serum/Plasma Kit (Qiagen, Hilden, Germany) according to the manufacturer’s instructions. Briefly, 1 µl of UniSp2 spike-in (Qiagen, Hilden, Germany), a control for the quality of RNA extraction, was combined with the lysis buffer before mixing with the serum. Total RNA (including miRNAs) was eluted in 14 µl of RNase-free water. Reverse transcription was performed using miRCURY LNA™ RT Kit (Qiagen, Hilden, Germany) according to the manufacturer’s instructions. Briefly, 1 µl of UniSp6 spike-in (Qiagen, Hilden, Germany), a control for the quality of RT reaction, was added to the reaction mix including 2 µl of total RNA, nucleic acid mix buffer and reverse transcriptase in a final volume of 10 µl. RT mix was incubated for 60 min at 42 °C and for 5 min at 95 °C. cDNA was stored at − 20 °C until analysis.

Expression value of hsa-mir-106b-3p, hsa-mir-143-3p, hsa-mir-144-3p, hsa-mir-150-5p, hsa-mir-18a-5p, hsa-mir-21-5p, hsa-mir-222-3p, hsa-mir-26a-5p, hsa-mir-335-5p, hsa-mir-361-3p, hsa-mir-375, hsa-mir-7-5p, and hsa-mir-942-5p was determined by real-time PCR using miRCURY LNA miRNA primers (Qiagen, Hilden, Germany) and miRCURY LNA™ SYBR Green PCR Kit (Qiagen, Hilden, Germany), with the instrument CFX384 (Biorad, USA). PCR cycling conditions were 95 °C for 2 min, 40 cycles of 95 °C for 10 s, 56 °C for 60 s and melting curve analysis 60–95 °C. The maximum cycle threshold (Ct) value was set at 280. UniSp2 and UniSp6 were used as control genes. Experiments were carried out in triplicates for each data point, and data analysis was done by using CFX Maestro Software (Biorad, USA). Data were expressed as relative expression using the 2-ΔΔCt method (compared to healthy patients).

## Results

### Samples selection, tissues morphological features and immunostaining

The patients’ cohort analyzed in the study was globally composed of 66 samples, including 29 GEP-NENs at different grades and 37 MTCs (Table 1 and Additional file 1: Table S1). Among them, 46 were either frozen tissues or FFPE and were used for the different assays, while 20 previously isolated RNA samples represented a MTC transcriptomics validation set. Moreover, for 5 GEP-NENs, we did not have access to primary tumors, but had instead metastatic tissues (2 hepatic and 3 lymphatic). Clinical and experimental information for each sample were summarized in Additional file 1: Table S1.

We applied a multi-omics experimental approach to identify altered genes, coding transcripts and miRNAs, specifically affecting functional pathways in the investigated NENs.

**Fig. 1:**
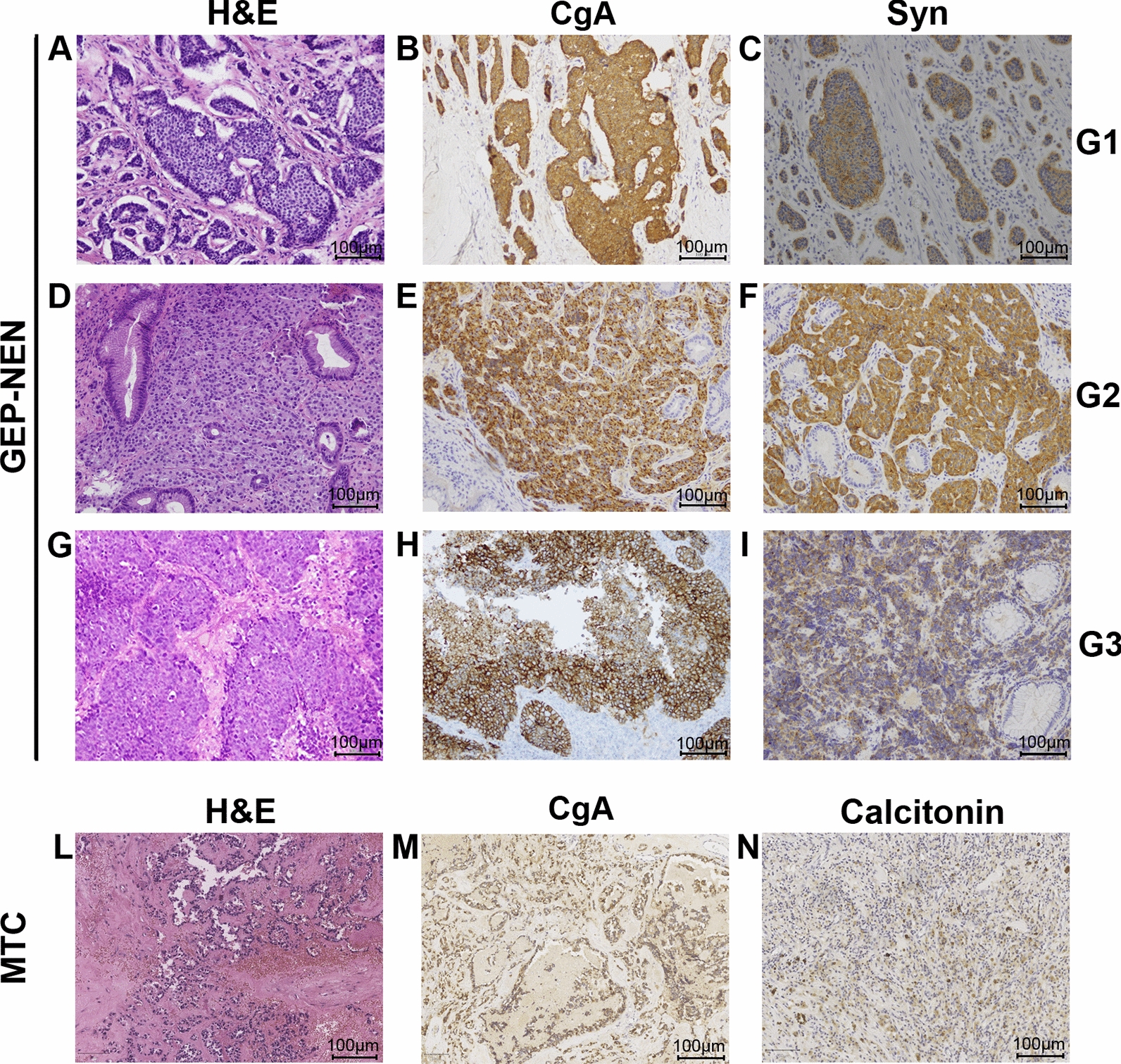
H&E and neuroendocrine markers expression in NEN tumors categories. **A** NETG1 H&E (×20); **B** positive CgA expression in NETG1 (20×); **C** positive Syn expression in NETG1; **D** NETG2 H&E; **E** positive CgA expression in NETG2(×20); **F** positive Syn expression in NETG2 (×20); **G** NECG3 H&E (×20); **H** positive CgA expression in NECG3 (×20); **I** positive Syn expression in NECG3(×20); **L** H&E in MTC (×20); **M** positive CgA expression in MTC (×20); **N** positive Calcitonin expression in MTC. Scale bar 100 µm

Where possible, according to the quality and the quantity of the samples available, we performed multigene panel sequencing, RNA-Seq and smallRNa-Seq (Additional file 1: Table S1). For a smaller set (30 samples), we also performed molecular karyotype by comparative genomics hybridization (CGH) array (Additional file 1: Table S1). Samples were classified as G1-G2 GEP-NETs, G3 GEP-NECs and MTCs, according to new histopathological classification, based on tumor grading and cell differentiation (Fig. 1A, D, G, L) [45, 46]. Moreover, we validated the positive staining of CgA and Syn immunohistochemical markers, used in routine histopathological diagnostics, to confirm tumor categories of NENs (Fig. 1B, E, H, C, F, I) [47] and the positive staining of CgA and Calcitonin, specific immunohistochemical markers of MTC (Fig. 1M, N) [48]. In Fig. 1 representative images of the above described morphologic and histopathologic features are shown. Clustering analysis, performed to evaluate possible samples variability emerging when comparing FFPE and fresh/frozen tissues, revealed that our samples were best grouped according to the histotype than to sample storage and library preparation method (data not shown). However, all these sources of variance were taken into account in downstream analyses.

### Assessment of multigene mutational signatures in NENs

**Fig. 2:**
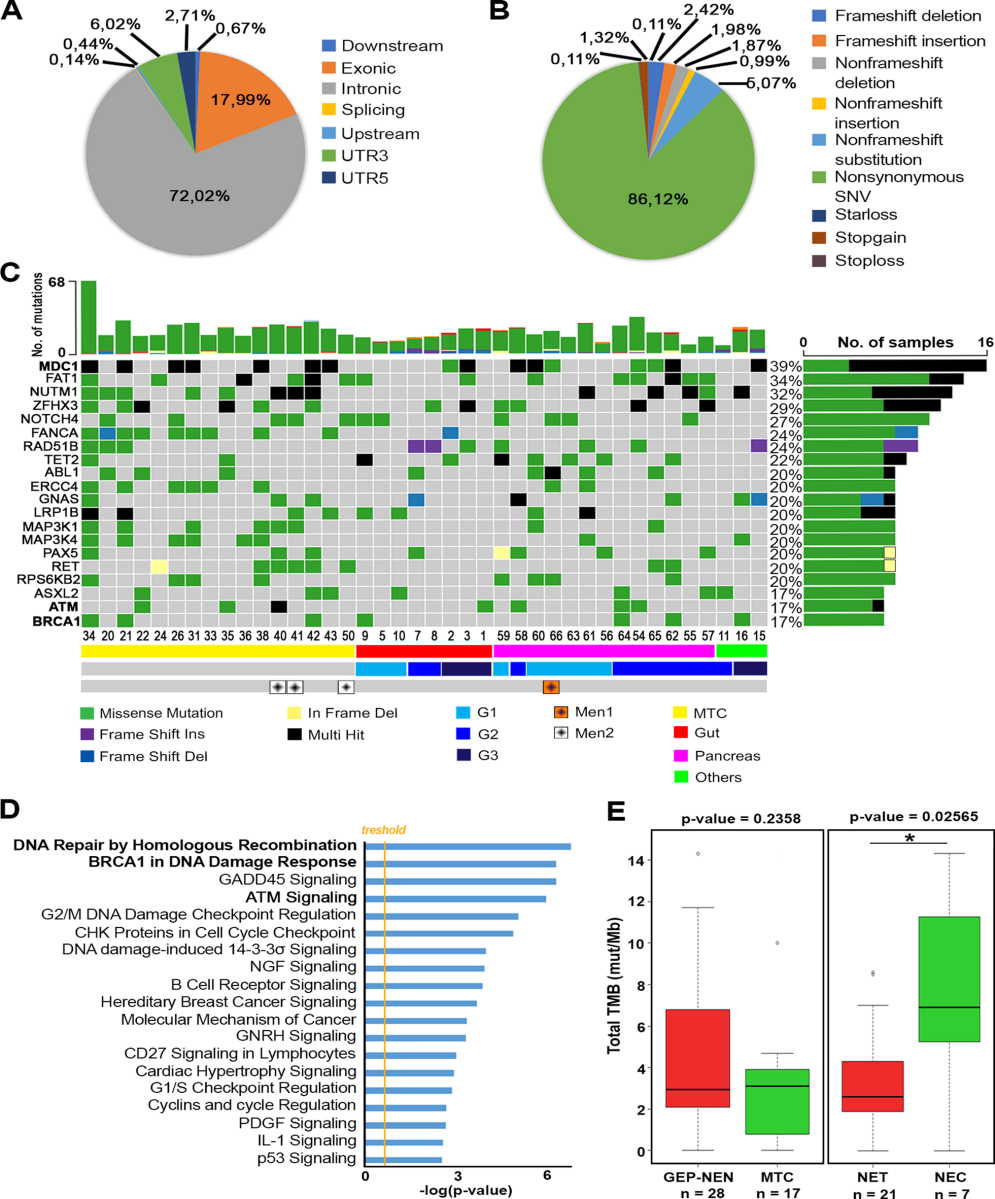
Mutational landscape of 44 NENs. **A** Pie chart showing proportions and genomic localizations of the identified variants after filtering and their **B** exonic function. **C** Oncoplot representation of the TOP 20 mutated genes in the analyzed samples. Only exonic and splicing mutations are represented. Each column represents individual patients with proper numeric code listed lower the graph and mutated genes are listed on the y-axis. The box colors indicate the type of mutation. The upper bar plots represent the total number of exonic/splicing variants identified for each sample within the represented gene set, while the right bar plots indicate the percentage of mutated samples for each gene. Colored bars in the bottom figure depict the pathological features of the patients; the upper is referred to cancer histotype: MTC, Gut, Pancreas and Others (lung-NEN and metastases), the middle indicates the WHO grade for GEP-NENs and the lower is referred to the presence of familiar syndrome. **D** Ingenuity Pathway Analysis (IPA) of the TOP 20 mutated genes. The red line indicates p-value threshold. **E** Boxplots showing Tumor Mutational Burden (TMB), as number of mutations per megabases, distribution comparing either GEP-NENs and MTCs (left panel) or NETs and NECs (right panel). T-test p-values are shown on the top

DNA isolated from 46 either frozen or FFPE NEN-tissues was analyzed by NGS, to identify genes more likely to be subject to sequence variations among 523 cancer-related ones. After sequencing, two samples were discarded from further analysis due to low quality. In detail, the method allows the simultaneous analysis of multiple biomarkers from different tumor tissues, assessing multiple variant types in a single assay, including small nucleotide variants (SNVs), indels, splice variants and emerging immunotherapy biomarkers such as tumor mutational burden (TMB) and microsatellite instability (MSI). This method has also been recently applied successfully for the identification of genomic features of head and neck neuroendocrine carcinoma [49]. In our case, all the sequence variants identified have been reported in Additional file 2: Table S2. After variants annotation and filtering out of the synonymous variants, the intergenic, those covered less than 100X, those present more than 5% within the general population, and those annotated as “benign” or “likely benign” in ClinVar database, as described in material and methods, we identified 5047 variants. Among them most were intronic, while near 18% were exonic (Fig. 2A); considering only exonic and splicing variants, more than 80% were nonsynonymous SNVs (Fig. 2B). In Fig. 2C are listed the top 20 mutated genes among all NENs examined, considering only exonic and splicing variants. Interestingly, the most frequently mutated gene among all kind of tissues analyzed was the mediator of DNA damage checkpoint protein 1 (MDC1). This is a critical DNA damage response (DDR) effector, acting as anti-apoptotic factor by interacting with TP53, whose loss is generally associated with genomic instability and tumorigenicity [50, 51]. Despite its key role in homologous recombination repair, also including other factors such as ATM and BRCA1 that are among the top mutated genes in the samples analyzed, it has never been found significantly altered in NENs so far, although its role in cancer development and treatment is emerging [52]. Another evidence was that, differently than most of the other genes, MDC1 was mostly affected by multiple variants per sample, a trend that was lesser shared by NUTM1, ZFHX3 and FAT1 respectively. Similar behavior was observed in single cases for the remaining, instead missense mutations were most frequently globally observed, while frameshift insertions or deletions and in frame deletions were sporadic events. Then, although we considered altogether all NEN samples, among the top 20 mutated genes NUTM1, ERCC4, MAP3K1, MAP3K4, RET and RPS6KB2, differently from the other genes, were never mutated in gut tissues but only in thyroid and pancreas deriving tumors (Fig. 2C). On the other hand, taking into account MTCs and GEP-NENs independently (Additional file 3: Fig. S1), we retrieved, within the top mutated genes, already known drivers and aggressiveness markers, such as HRAS and RET for MTCs (Additional file 3: Fig. S1A), MEN 1 for GEP-NETs (Additional file 3: Fig. S1B), RB1 and TP53 for GEP-NECs (Additional file 3: Fig. S1C).

Functional annotation analysis performed on the globally top mutated genes confirmed, as expected, that these are involved in pathways related to genome stability maintenance, such as DNA repair by homologous recombination, DNA damage response and checkpoint regulation and ATM signaling (Fig. 2D). Finally, analyzing TMB status considering GEP-NEN and MTC groups, the median value was around 3; when considering NETs and NECs separately, within the GEP-NEN group, the median value increased significantly in NECs, in some cases exceeding 10 that is the threshold value over which a subject is predicted to be responsive to immunotherapy (Fig. 2E). MSI quantitative score, instead, was not informative, in line with what observed by others (data not shown).

**Fig. 3:**
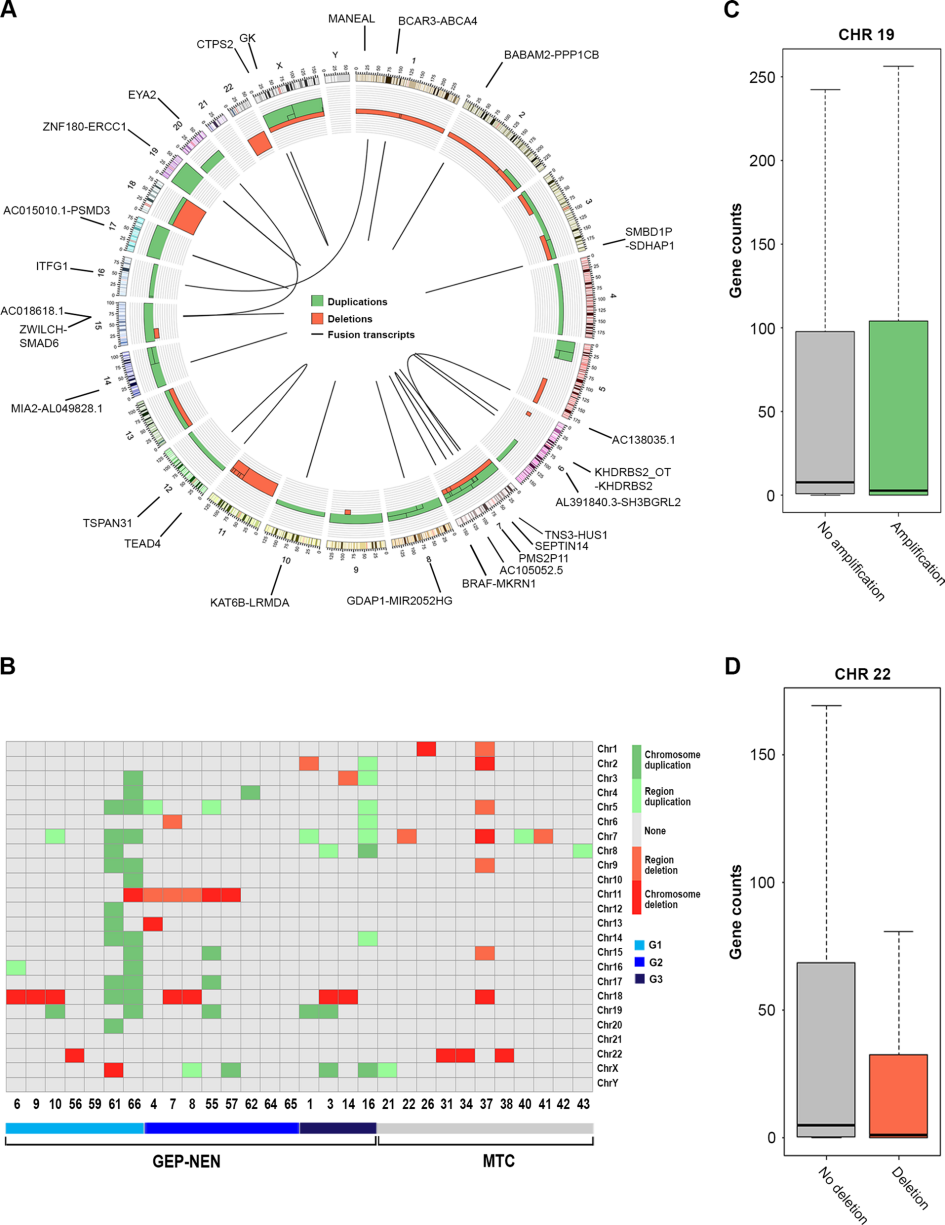
Genomic rearrangements of NENs. **A** Circos plot showing aCGH-derived genomic rearrangements (middle ring) and fusion transcripts (inner ring) retrieved among the analyzed samples. External colored ring indicates chromosomes. Red bars represent deletions, while green ones show amplifications. The grid behind the bars shows the number of samples presenting rearrangements. The thin lines in the bars represent the presence of only a part of the aberration in some samples. The curved lines in the inner ring indicate the fusion genes in their exact location. Gene names are shown on the outer ring. **B** Heatmap representing aCGH-derived genomic rearrangements in the 30 samples considered for analysis. Each raw represents a chromosome while each column represents a patient whose numeric code is listed in the lower side. Shades of red and green indicate the rearrangement type as shown in the side legend. The bar with the different color blue shades represents tumor grades. **C** Boxplot representing gene counts between samples showing (green) or lacking (grey) amplification of chromosome 19. **D** Boxplot representing gene counts between samples having (red) or not (grey) deletion of chromosome 22

DNA from 30 out of the 44 above analyzed samples was also subjected to molecular karyotype by CGH array, to evaluate the presence of CNVs. At a first glance the intriguing result was that, while GEP-NENs in general presented multiple deletions/duplications and several mosaicisms, with chromosomes 5, 6, 7, 11, 18, 22 and X mainly affected, MTCs mostly showed single events per sample, except for one case, mostly representing deletions (Fig. 3A, B). From the previous analysis of the multigene panel test that allows the identification of gene amplifications, it emerged that EGFR and BRAF genes, both located on chromosome 7, were among the most frequently amplified in our GEP-NEN samples (Table 2). The most commonly amplified of all, RPS6KB1, is instead located on chromosome 17 that resulted duplicated only in few cases (Table 2 and Fig. 3B). As expected, MTC samples did not show gene amplification events.

**Table 2:** Gene amplifications

Gene	Samples (n)	Sample codes	Average fold change
RPS6KB1	12	1, 3, 5, 7, 8, 10, 11, 12, 15, 16, 55, 56	1.6
EGFR	9	2, 5, 8, 13, 15, 60, 61, 63, 65	1.6
BRAF	6	1, 5, 12, 13, 15, 61	1.5
ALK	4	5, 8, 10, 13	1.5
ERBB2	4	5, 12, 13, 55	1.9
FGF1	4	2, 4, 60, 61	1.6
FGFR1	4	2, 5, 13, 16	4.5
CCND3	3	2, 10, 16	1.5
CDK4	3	3, 61, 63	9.1
ERCC2	3	3, 5, 55	1.9
FGF10	3	3, 60, 61	1.7
FGF7	3	3, 15, 55	1.4
KIT	3	12, 13, 65	1.5
PDGFRA	3	5, 8, 12	1.5
RAF1	3	2, 5, 13	1.6
ERCC1	2	3, 5	2.0
FGF2	2	8, 12	1.5
LAMP1	2	5, 61	1.6
MET	2	13, 61	1.5
PDGFRB	2	60, 61	1.7
PIK3CA	2	5, 16	1.7
AR	1	3	1.5
BRCA2	1	2	1.4
CCND1	1	63	10.6
CDK6	1	61	1.6
ERBB3	1	63	2.8
FGF19	1	63	8.8
FGF23	1	3	1.5
FGF3	1	63	10.3
FGF4	1	63	10.3
FGFR2	1	5	2.1
FGFR4	1	61	1.9
JAK2	1	2	2.5
KRAS	1	3	1.4
MDM2	1	63	5.6
MDM4	1	15	1.5
MYC	1	3	26.5
MYCL	1	16	4.0
RICTOR	1	61	1.7
TFRC	1	5	3.1

Since total RNA was also sequenced by NGS for the same sample set as above, plus 20 more for which only RNA was available (Additional file 4: Table S3), data was analyzed to assess the concordance of gene expression data with the amplification/deletion patterns described and for the presence of fusion transcripts. Regarding the first point, significant correspondence between DNA rearrangements and gene counts was observed in most cases. This was relieved, among others, for chromosome 19, where patients harboring amplifications showed a trend to an overall increased gene counts within the same chromosome with respect to not amplified samples (Fig. 3C), and for chromosome 22, in which an opposite scenario was observed for patients showing deletions (Fig. 3D). For the second point, according to the filtering criteria described in the methods and after discarding low quality samples (6 out of the 66 samples available), 20 fusion transcripts were detected. Most of them have been found only in 1 patient and few in 2 or 3 patients (Table 3, Fig. 3A). Interestingly, although MTC tissues were very little subject to the presence of fusion transcripts, confirming the low frequency of chromosomal rearrangement events in this type of neoplasm, the fusion AL391840.3-SH3BGRL2, previously identified in high-grade serous ovarian cancer [53], was retrieved in two MTC patients in our cohort (Table 3). Further experiments will be needed to investigate the functional significance of these rearrangements in NENs development and patients’ survival.

**Table 3:** Fusion transcripts

Fusion_name	Samples (n)	Samples code	Junction_Readcount
PMS2P11-AC105052.5	3	15, 57, 59	10_20_21
AL391840.3-SH3BGRL2	2	27, 37	10_12
GDAP1-MIR2052HG	2	65, 66	10_15
MANEAL-ITFG1	2	15, 57	87_127
AC138035.1-SEPTIN14	1	65	16
BCAR3-ABCA4	1	13	17
CTPS2-GK	1	3	24
KHDRBS2_OT-KHDRBS2	1	57	17
LRMDA-KAT6B	1	13	12
MIA2-AL049828.1	1	61	11
MKRN1-BRAF	1	30	13
PMS2P9-AC105052.5	1	66	12
PPP1CB-BABAM2	1	13	11
PSMD3-AC015910.1	1	13	12
SMAD6-ZWILCH	1	13	57
SMBD1P-SDHAP1	1	39	11
TNS3-HUS1	1	13	38
TSPAN31-TEAD4	1	3	226
ZNF180-ERCC1	1	13	17
ZNF609-EYA2	1	13	72

### NEN miRNome profiling

**Fig. 4:**
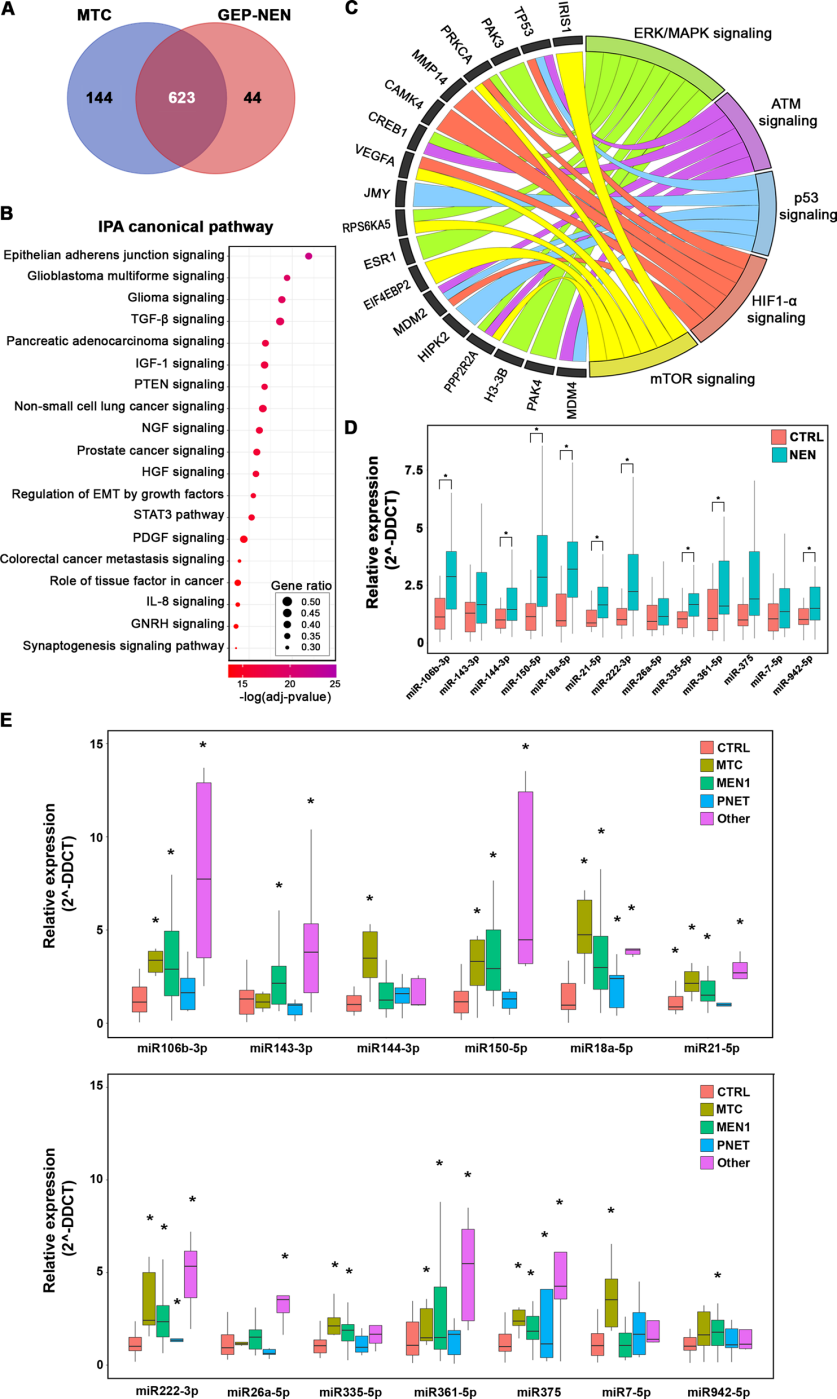
miRNA profiling in NENs tissue and serum. **A** Venn Diagram comparing MTC and GEP-NEN expressed miRNAs. **B** Dot plot of the most significant IPA canonical pathways involving commonly expressed miRNAs. Dot color ranges from red to purple depending on the –log of the adjusted p-value. Dot sizes depend on the Gene Ratio as described in the figure. **C** GO-Plot showing some of the most important pathways involving the predicted target genes of miRNAs selected for validation. Different pathways are drawn with different colors. The pathway-involved target genes are reported in the outer ring. **D** Boxplots of the relative expression (2^-DDCT) of each serum validated miRNA in NEN (blue) samples versus healthy controls (red). Asterisks indicate statistical significance (p-value < 0.05). **E** Boxplots of the relative expression (2^-DDCT) of each serum validated miRNA in different samples groups mentioned in the figure. The asterisks indicate statistical significance (p-value < 0.05)

To investigate miRNA expression profiles with the attempt to identify deregulated molecules useful as NEN biomarkers, smallRNA-Seq was performed (Additional file 5: Table S4), allowing the identification of 623 miRNAs commonly expressed in GEP-NENs and in MTCs (Fig. 4A).

Functional analysis on their target mRNAs, expressed in the investigated samples (Additional file 6: Table S5), revealed their involvement in pathways implicated in neurologic and endocrine cancers, such as glioblastoma multiforme, glioma and pancreatic adenocarcinoma signaling, together with others specifically involved in tumor development and progression, including epithelial adherens junctions, TGF-β, PTEN and NGF signalings (Fig. 4B). Lacking either paired or independent normal tissues, it was not possible to identify differentially expressed coding and noncoding transcripts in cancer samples. Despite this, considering only GEP-NEN tissues, we evaluated the possible evidence of differentially expressed miRNAs among grades and this was indeed the case. How other research groups already hypothesized [54], a set of miRNAs, 52 in our case, were significantly differentially expressed between NETs and NECs and some of them also seemed to discriminate tumor grades (Additional file 7: Fig. S2). To identify possible circulating NENs biomarkers, smallRNA-Seq was performed on 3 patients for whom both tissues and serum samples were available; the same tissues underwent multi omics analyses previously. This allowed the identification of 186 and 176 miRNAs respectively in two MTC sera and 232 in one pancreatic NET serum; from 94 to 98% of them was also expressed in the corresponding tissues (Additional file 5: Table S4). Then, a set of 13 miRNAs was selected for validation, taking into account the top100 expressed in the tissues analyzed, differentially expressed ones between NETs and NECs, as well as published evidences. The validation cohort was composed of both MTC and GEP-NEN patients; the former were further distinguished in either sporadic or hereditary (MEN1). Controls were globally matched for age and gender and, totally, 34 healthy controls and 42 patients were enrolled; among patients only 2 were G3 NENs, thus making not possible GEP-NEN grade stratification according to secreted miRNAs. Considering specifically the mRNAs targeted by the selected miRNA subset (Additional file 8: Table S6), these are implicated in ERK/MAPK, mTOR, HIF-1α, p53 and ATM signal transduction pathways (Fig. 4C). Some of these pathways, in particular p53 and ATM, were resulted also affected by gene variants (Fig. 2D), thus suggesting a multi-level deregulation of such cascades during NENs development and progression. As shown in Fig. 4D, all the selected miRNAs resulted to be more highly expressed in patients with respect to controls, most of them significantly discriminating between tumor affected and healthy subjects. On the other hand, grouping patients sera according to the origin of NEN, namely thyroid (MTC), pancreas sporadic (pNEN), pancreas familial (MEN1) or others (mostly including gut and lung NENs), it emerged that some miRNAs were significantly deregulated in specific subgroups. Specifically, miR143-3p was significantly upregulated in MEN1 and other NENs, miR144-3p and miR7-5p in MTC, miR335-5p in MTC and MEN1 and miR942-5p in MEN1. (Fig. 4E). Instead, miR-375 was significantly up-regulated in all the different subgroups and it was already proposed as circulating biomarkers in other cancer types [55]. However, this effect might be due to subgroups relative size and a larger case series would be needed to confirm the data.

### Clinically actionable pathways prediction

**Fig. 5:**
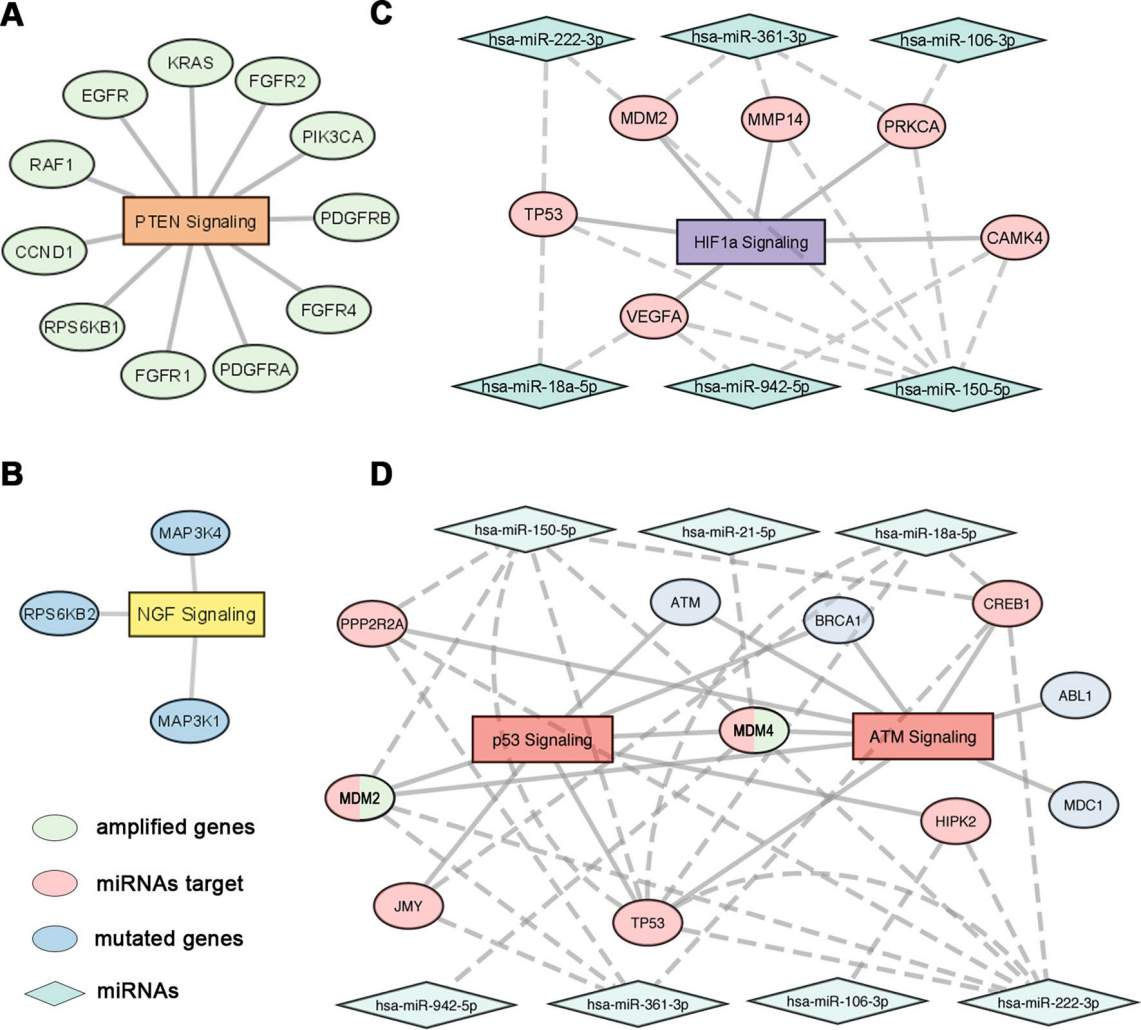
Functional interaction networks. **A** Network of amplified genes found through multigene panel sequencing. **B** Functional network involving only mutated genes. **C** HIF1a signaling network involving miRNA targets. **D** Multi-level network considering both miRNA targets and gene variations. Circles are labeled with gene names, rectangles represent pathways, while diamonds the miRNAs. Different colors of the circles indicate if the genes are amplified (green), mutated (blue), targeted by miRNA (pink) or targeted and/or amplified (pink and green). Dashed lines indicate correlation between miRNAs and their targets, while full lines indicate gene connections to specific signalings

In the investigated NENs, multi-omics analysis has revealed frequently mutated and duplicated genes, chromosome duplications/deletions, fusion transcripts and deregulated miRNAs. Altogether, considering this data, functional analysis was performed to highlight the most affected pathways that may be useful therapeutic targets in the treatment of these neoplasms. In details, considering the most frequently amplified genes in our dataset, PTEN signaling emerged among the most significantly affected (Fig. 5A) as already known especially for pancreatic NENs [11, 12]. In addition, the NGF signaling (Fig. 5B) known to be implicated in neuroendocrine neoplasms [56], includes some of the most frequently mutated genes in our casuistry. On the other hand, among deregulated miRNA targets, in Fig. 5C we reported those miRNAs significantly enrolled in HIF1α signaling (Fig. 5C), known to be activated by RET in MTCs [57]. Considering, instead, the pathways impacted at different levels by gene alterations and miRNA deregulation, the well described p53 and the newly associated ATM signaling emerged among those significantly enriched, the former representing a potential novel therapeutic target in the treatment of this class of cancers. Indeed, the last network, showed in Fig. 5D, comprises deregulated miRNA targets, mutated genes and, among them, MDM2 and MDM4 represent both downstream miRNA targets that have also been retrieved as amplified genes in some of the analyzed tissues, reinforcing the hypothesis of a possible multi-level targeting.

## Discussion

NENs represent a heterogeneous group of neoplastic diseases originating from neuroendocrine cells distributed throughout the body. Conventionally, this cancer types are considered very difficult to diagnose due to the lack of molecular and prognostic markers and the difficulties in identifying the primary site of origin. Being mostly indolent, they are often already metastatic at diagnosis [3] and, indeed, selected NEN specimens are being included in clinical studies aiming at tumor origin identification [58]. Thus, the identification of early and specific diagnostic and prognostic markers as well as novel therapeutic targets is crucial. The current landscape of NENs genetic knowledge is heterogeneous, with well-defined traits by high-throughput studies for some anatomic sites such as pancreatic NENs, but relatively low information for other sites. Moreover, the increasing interest in circulating biomarkers offers a new perspective for earlier NENs diagnosis [59]. Furthermore, some studies have been conducted to emphasize the potential of miRNAs as biomarkers and for easier grade stratification and tissue discrimination in GEP-NENs [54, 60–62] and as promising diagnostic and prognostic factors in MTC [63–65].

In the present paper, a multi-omics approach has been applied to analyze a diversified NENs cohort composed of GEP-NENs and MTC primary tumors and few GEP-NEN derived metastasis. Given the heterogeneity of the cohort, with a small sample size for each group making it difficult to identify a histotype specific molecular signature, and considering the recently emerged concept that NENs from different organ systems inter-relate clinically and genetically [4], we focused on highlighting common molecular and functional features that might represent useful and effective NEN biomarkers and therapeutic targets.

The gene mutational landscape revealed, over the already known key drivers specifically associated with pancreatic NETs, pancreatic NECs [11, 66] and MTCs [6], a common signature represented by a set of genes most frequently mutated, among which those involved in genome stability maintenance and DNA damage recombination emerged, including MDC1, BRCA1 and ATM. In particular, MDC1 was never specifically associated to NENs before, except for one paper observing the presence of either MDC1 or ATM somatic mutations in RET and RAS negative MTCs [67]. In our cohort, instead, MDC1 resulted the top mutated gene in absolute among all NEN specimens analyzed. It is a key component of the DNA damage response, binding to γ-H2AX at DNA double-strand breaks, and participating in the recruitment of key factors including ATM, BRCA1, and TP53 [68]. MDC1 loss of function could negatively affect both homologous recombination and non-homologous end joint repair pathways and co-mutation with some of its key co-factors has been proposed as potential marker for radiosensitivity [69]. Interestingly, within our cohort MDC1 resulted to be co-mutated with ATM in 2 out of 7 ATM-mutated samples and with BRCA1 in 4 out of 7 BRCA1-mutated ones. Moreover, we also found one ATM-BRCA co-mutation case (Fig. 2C). With these premises, not surprisingly, ATM signaling resulted among the most significantly impacted at gene level, together with other pathways involving BRCA and DNA repair, or enrolling ATM downstream targets such as G2/M and G1/S checkpoints and other DNA damage-induced ones (Fig. 2D) [70]. On the other hand, complementary analyzes carried out on DNA and RNA samples, revealed a different behavior of GEP-NENs and MTCs regarding the tendency to form large chromosome rearrangements or gene amplifications and fusion transcripts. Indeed, as also observed before, GEP-NENs were more prone to this kind of events. Moreover, we observed a good concordance between the amplification/deletion patterns observed at chromosome level and gene expression data (Fig. 3). Given the absence of matched normal tissues, we did not sought to investigate differential expressed genes in tumor tissues except to have an indication of a possible deregulation among GEP-NENs of different grades even if this was not the main focus of the present work. As expected, several transcripts were found differentially expressed between grades, functionally targeting most of the pathways observed to be affected at the gene level, and, thereafter, post-transcriptionally through miRNA targeting (data not shown). Indeed, in the attempt to mainly focus on molecular features in common to the analyzed neoplasms, we proceeded by analyzing tissue miRNAs to identify those over-expressed in the various kind of investigated NENs. This led to the identification of a set of 623 commonly expressed miRNAs, with the top expressed being also mostly shared among different samples. Anyway, even in this case, a set of miRNAs resulted to be expressed at different level in low with respect to high-grade GEP-NENs, thus corroborating previous findings pointing to miRNAs as useful biomarkers for grade stratification in this class of NENs [54, 61]. In this context, considering our and others’ evidence of high TMB in high-grade GEP-NENs and the proposed role for PDL1 expression in GEP-NENs grade stratification [71], a positive response of NETs-G3 and NECs to immunotherapy may be desirable and the impact of miRNA-mediated deregulation on PDL1 and other genes involved in the immunological synapse should be deeply investigated. In our case, although we observed PDL1 coding transcript over-expression in G3 vs G2 tumors, this appears not to be dependent on miRNA-mediated post-transcriptional regulation, although the limited number of high-grade tumors may have undermined the significance of the results (data not shown). Moreover, through a pilot sequencing by NGS of serum miRNAs from 3 patients, multiple miRNA molecules could be detected, with more than 95% of them being previously identified in the corresponding tissues. This finding reinforced the hypothesis that overexpressed miRNAs may be specifically released and evaluated as circulating NEN biomarkers.

Based on these results, we selected a set of 13 miRNAs to be evaluated in serum samples as possible circulating NEN biomarkers. We found that these miRNAs were overall significantly overexpressed in NEN patients compared to healthy subjects. This result represents a remarkable one, because for the first time a set of circulating miRNAs overexpressed in NENs patients could potentially represent a pathological signature for diagnostic purposes. A larger cohort with higher sample number for each histotype would be needed to confirm the data and select the most suitable molecule combination to be assessed for specific and reliable results.

Very interestingly, the mRNA targets of the selected miRNA panel are linked to ATM signaling (Fig. 4D) that we found emerging among the most significantly impacted at multiple levels (Fig. 5D). Taken together, we can speculate that ATM may represent a novel druggable pathway, in addition to the widely used inhibitors of mTOR [72–74], whose regulation by ATM has been also demonstrated [75, 76].

Indeed, following the success of PARP inhibitors, ATM inhibitors have been proposed in the therapeutic exploitation of DNA Damage Response (DDR) in cancer [77]. Several synthetic molecules have been already developed and demonstrated to induce significant sensitization to radiation and DNA-damaging chemotherapeutic agents [78], and some of them are undergoing clinical trials in combination with radiation therapy [79] or with PARP inhibitors [80].

In fact, according to an experimentally proven hypothesis, the use of an ATM inhibitor, shutting down the MDC1-mediated DDR pathway, together with PARP inhibitors, which rescue endogenous BIN1 expression (that increases cell death due to DNA damage), is able to generate a new ‘BRCAness-independent’ synthetic lethal effect in cancerous cells [81].

Taken together, our results reinforce this hypothesis, but further experimental and preclinical evidences are needed by establishing in vitro and in vivo models demonstrating their effectiveness and potential clinical application in NENs.

## Conclusions

The findings of the present study highlighted a novel molecular landscape of NENs, allowing the identification of a set of circulating miRNAs that may be investigated as NEN biomarkers, and suggesting ATM and its cofactors as possible molecular targets to be tested in combination with current therapies.

## Supplementary Information

**Additional file 1: Table S1.** Sample information.

**Additional file 2: Table S2.** TSO500 all variants.

**Additional file 3: Fig. S1.** Mutational profile of different NEN classes. (A) Oncoplot representation of the TOP 20 mutated genes in MTC samples. The gray bar in the bottom figure highlights the presence of familiar syndrome MEN2. (B) Oncoplot representation of the TOP 20 mutated genes in GEP-NETs (G1 and G2 NENs). Gray bars at the bottom indicate, the presence of familiar syndrome MEN1 (upper) and metastatic tissues (lower). (C) Oncoplot representation of the TOP 20 mutated genes in GEP-NEC. The bottom bar indicates metastases. In each graph each column represent a sample with its numeric code while each raw represent a gene. Box colors refer to mutation type.

**Additional file 4: Table S3.** RNA_Seq all counts.

**Additional file 5: Table S4.** SmallRNA normalized counts.

**Additional file 6: Table S5.** Targets of common miRNAs.

**Additional file 7: Fig. S2.** Heatmap of Differentially Expressed miRNAs between GEP-NETs (G1 and G2) and GEP-NEC plus metastases (G3). The log transformed median for each group is shown.

**Additional file 8: Table S6.** Targets of miRNAs selected for validation.

## Data Availability

The datasets supporting the findings of this study are available from the corresponding author upon reasonable request. The Raw sequencing data have been deposited in the EBI ArrayExpress database (http://www.ebi.ac.uk/arrayexpress) with the following accession numbers: E-MTAB-11529 for multigene panel sequencing; E-MTAB-11599 for RNA-Seq and E-MTAB-11598 for smallRNA-Seq data.

## Abbreviations

NEN: Neuroendocrine neoplasm
GEP-NEN: Gastroenteropancreatic neuroendocrine neoplasm
MTC: Medullary thyroid carcinoma
ATM: Ataxia Telangiectasia Mutated
MEN1: Multiple endocrine neoplasia type 1
MEN2: Multiple endocrine neoplasia type 2
VHL: Von Hippel–Lindau
NF1: Neurofibromatosis type 1
TSC: Tuberous sclerosis complex
WHO: World Health Organization
IARC: International Agency for Research on Cancer
NET: Neuroendocrine tumor
NEC: Neuroendocrine carcinoma
DAXX: Death Domain Associated Protein
ATRX: Alpha thalassemia/mental retardation syndrome X-linked
PTEN: Phosphatase and tensin homolog
TSC2: Tuberous sclerosis complex 2
mTOR: Mammalian target of rapamycin
MUTYH: MutY DNA glycosylase
CHEK2: Checkpoint Kinase 2
BRCA2: BReast CAncer gene 2
GI-NET: Gastrointestinal neuroendocrine tumor
CDNK1B: Cyclin dependent kinase inhibitor 1B
KRAS: Kirsten rat sarcoma virus
SMAD4: SMAD family member 4
TP53: Tumor Protein P53
BRCA1: BReast CAncer gene 1
RB1: Retinoblastoma-associated protein
RET: Ret proto-oncogene
RAS: Rat sarcoma virus
CgA: Chromogranin A
Syn: Synaptophysin
5-HIAA: 5-Hydroxyindoleacetic acid
NSE: Neuron-specific enolase
CD56: Cluster of differentiation 56
FFPE: Formalin fixed paraffin embedded
H&E: Hematoxylin and eosin
DIN: DNA integrity number
RIN: RNA integrity number
TMB: Tumor mutational burden
MSI: Microsatellite instability
IPA: Ingenuity pathway analysis
Ct: Cycle threshold
CGH: Comparative genomics hybridization
NGS: Next generation sequencing
SNV: Small nucleotide variant
CNV: Copy number variation
MDC1: Mediator of DNA damage checkpoint protein 1
DDR: DNA damage response
NUTM1: NUT midline carcinoma family member 1
ZFHX3: Zinc finger homeobox 3
FAT1: FAT atypical cadherin 1
ERCC4: Excision repair cross-complementation group 4
MAP3K1: Mitogen-activated protein kinase kinase kinase 1
MAP3K4: Mitogen-activated protein kinase kinase kinase 4
RPS6KB2: Ribosomal protein S6 kinase beta-2
HRAS: HRas proto-oncogene
EGFR: Epidermal growth factor receptor
BRAF: B-Raf proto-oncogene
TGF-β: Transforming growth factor beta
NGF: Nerve growth factor
ERK: Extracellular signal-regulated kinase
MAPK: Mitogen-activated protein kinase 1
HIF-1α: Hypoxia inducible factor 1 subunit alpha
pNEN: Pancreas sporadic neuroendocrine neoplasm
MDM2: MDM2 proto-oncogene, E3 ubiquitin protein ligase
MDM4: Mdm2-like P53-binding protein
γ-H2AX: H2A.X variant histone
PARP: Poly(ADP-ribose) polymerase 1
