# Supplementary material for: Identification of functional pathways and molecular signatures in neuroendocrine neoplasms by multi-omics analysis
Source: J Transl Med. 2022 Jul 6;20:306. doi: 10.1186/s12967-022-03511-7 (PMC9258165; doi:10.1186/s12967-022-03511-7)
Supplement: Supplementary file 3 — Additional file 3: Fig. S1. Mutational profile of different NEN classes. (A) Oncoplot representation of the TOP 20 mutated genes in MTC samples. The gray bar in the bottom figure highlights the presence of familiar syndrome MEN2. (B) Oncoplot representation of the TOP 20 mutated genes in GEP-NETs (G1 and G2 NENs). Gray bars at the bottom indicate, the presence of familiar syndrome MEN1 (upper) and metastatic tissues (lower). (C) Oncoplot representation of the TOP 20 mutated genes in GEP-NEC. The bottom bar indicates metastases. In each graph each column represent a sample with its numeric code while each raw represent a gene. Box colors refer to mutation type. [file 12967_2022_3511_MOESM3_ESM.docx]

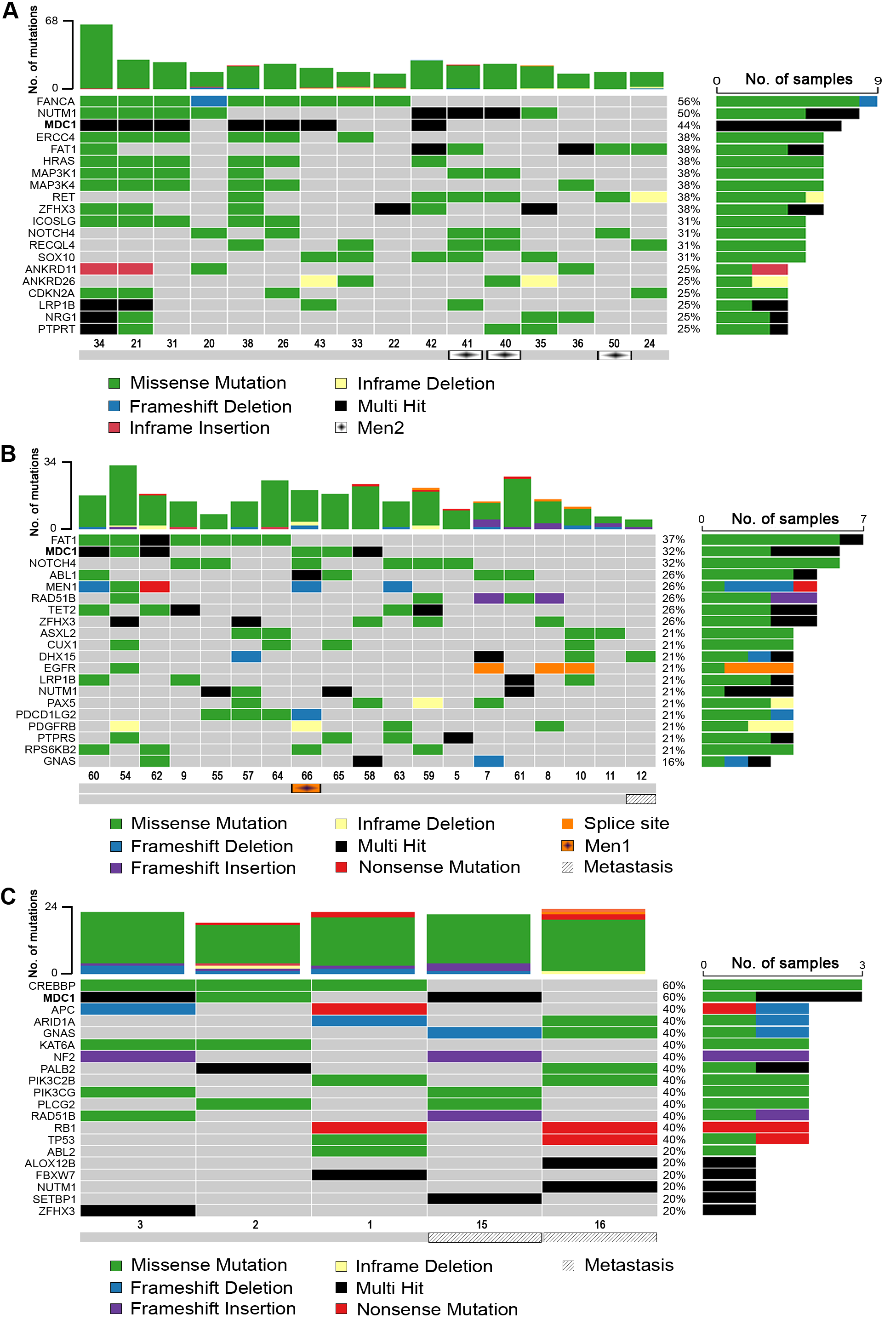


**Fig. S1:** Mutational profile of different NEN classes. (A) Oncoplot representation of the TOP 20 mutated genes in MTC samples. The gray bar in the bottom figure highlights the presence of familiar syndrome MEN2. (B) Oncoplot representation of the TOP 20 mutated genes in GEP-NETs (G1 and G2 NENs). Gray bars at the bottom indicate, the presence of familiar syndrome MEN1 (upper) and metastatic tissues (lower). (C) Oncoplot representation of the TOP 20 mutated genes in GEP-NEC. The bottom bar indicates metastases. In each graph each column represent a sample with its numeric code while each raw represent a gene. Box colors refer to mutation type.
