# Supplementary material for: Identification of functional pathways and molecular signatures in neuroendocrine neoplasms by multi-omics analysis
Source: J Transl Med. 2022 Jul 6;20:306. doi: 10.1186/s12967-022-03511-7 (PMC9258165; doi:10.1186/s12967-022-03511-7)
Supplement: Supplementary file 7 — Additional file 7: Fig. S2. Heatmap of Differentially Expressed miRNAs between GEP-NETs (G1 and G2) and GEP-NEC plus metastases (G3). The log transformed median for each group is shown. [file 12967_2022_3511_MOESM7_ESM.docx]

**
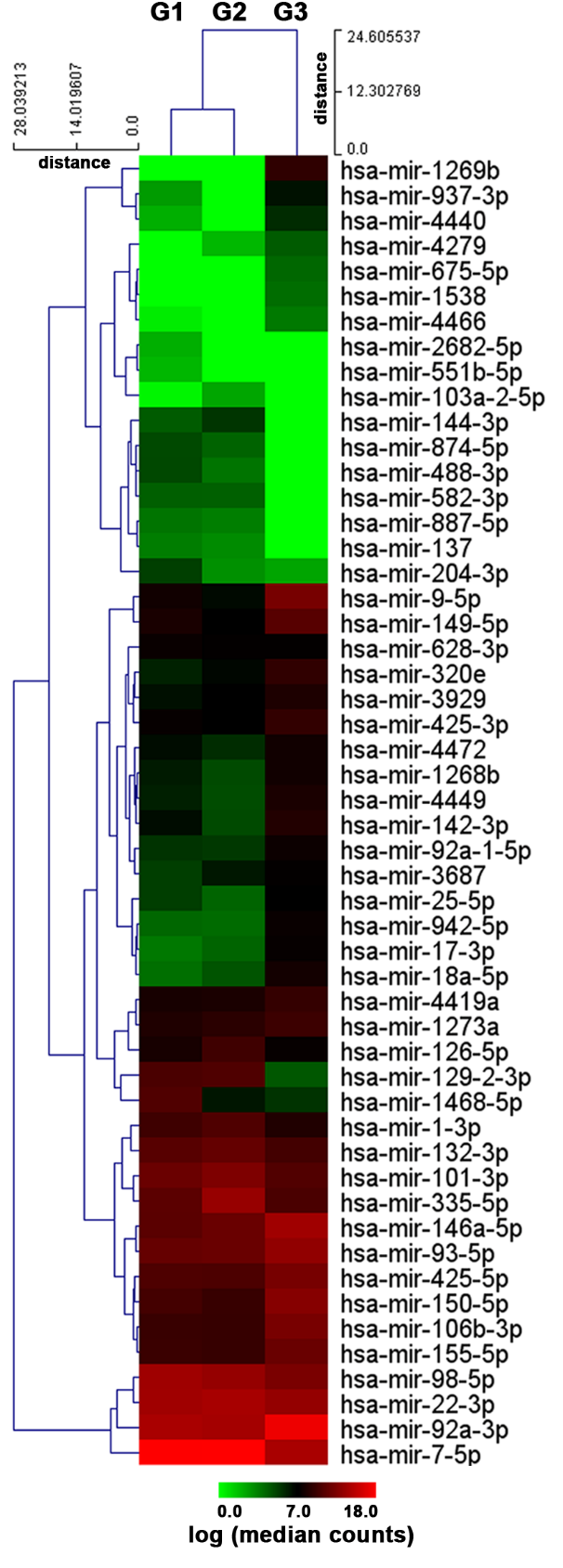
**

**Fig. S2:** Heatmap of Differentially Expressed miRNAs between GEP-NETs (G1 and G2) and GEP-NEC plus metastases (G3). The log transformed median for each group is shown.
